# Study of lncRNAs in Pediatric Neurological Diseases: Methods, Analysis of the State-of-Art and Possible Therapeutic Implications

**DOI:** 10.3390/ph16111616

**Published:** 2023-11-16

**Authors:** Cecilia Pandini, Federica Rey, Cristina Cereda, Stephana Carelli, Paolo Gandellini

**Affiliations:** 1 Department of Biosciences, University of Milan, 20133 Milan, Italy; cecilia.pandini@unimi.it; 2 Pediatric Clinical Research Center “Fondazione Romeo ed Enrica Invernizzi”, Department of Biomedical and Clinical Sciences, University of Milan, 20157 Milan, Italy; federica.rey@unimi.it (F.R.); stephana.carelli@unimi.it (S.C.); 3 Center of Functional Genomics and Rare Diseases, Department of Pediatrics, Buzzi Children’s Hospital, 20157 Milan, Italy; cristina.cereda@asst-fbf-sacco.it

**Keywords:** LncRNAs, brain development, pediatric brain cancer, neurodevelopmental disorders, pediatric neural degeneration

## Abstract

Long non-coding RNAs (lncRNAs) have emerged as crucial regulators in various cellular processes, and their roles in pediatric neurological diseases are increasingly being explored. This review provides an overview of lncRNA implications in the central nervous system, both in its physiological state and when a pathological condition is present. We describe the role of lncRNAs in neural development, highlighting their significance in processes such as neural stem cell proliferation, differentiation, and synaptogenesis. Dysregulation of specific lncRNAs is associated with multiple pediatric neurological diseases, such as neurodevelopmental or neurodegenerative disorders and brain tumors. The collected evidence indicates that there is a need for further research to uncover the full spectrum of lncRNA involvement in pediatric neurological diseases and brain tumors. While challenges exist, ongoing advancements in technology and our understanding of lncRNA biology offer hope for future breakthroughs in the field of pediatric neurology, leveraging lncRNAs as potential therapeutic targets and biomarkers.

## 1. Introduction

### 1.1. Long Non-Coding RNAs

Long non-coding RNAs (lncRNAs) represent a diverse and expanding class of RNA molecules longer than 200 nucleotides that do not encode for proteins, but nevertheless play essential roles in various cellular processes [1]. Several classification criteria are commonly employed to organize the complex lncRNA world; most of these classifications are not mutually exclusive and are in constant evolution [2]. Based on their genomic localization, lncRNAs can be classified into: intergenic lncRNAs, located between protein-coding genes; intronic lncRNAs, residing within introns of protein-coding genes; antisense lncRNAs, transcribed from the antisense strand of protein-coding genes, and bidirectional lncRNAs, produced from the same promoter of protein-coding genes on the opposite strand [3].

LncRNAs exert their functional role through a diverse array of mechanisms that contribute to the regulation of gene expression and cellular processes [1,2,3,4,5]. Firstly, lncRNAs can act as molecular scaffolds, facilitating the assembly of protein complexes by physically interacting with multiple binding partners. This scaffolding role enables lncRNAs to orchestrate the spatial organization of regulatory complexes, influencing the activation or repression of specific genes. Additionally, lncRNAs often function as guides or decoys by directly binding to proteins or other nucleic acids, thus modulating their activity or availability. Through these mechanisms, lncRNAs can finely tune signaling pathways or compete for binding sites, thereby shaping the cellular response to environmental cues. Moreover, lncRNAs are frequently engaged in transcriptional regulation by modulating chromatin architecture and epigenetic modifications. Acting as epigenetic regulators, lncRNAs can recruit chromatin-modifying enzymes to specific genomic loci, leading to alterations in DNA methylation, histone acetylation/methylation, and chromatin accessibility. This influence on chromatin states subsequently affects gene expression patterns, contributing to cell fate determination and differentiation processes [1,3,5]. Another prominent mechanism through which lncRNAs operate is post-transcriptional regulation [2,5,6]. By interacting with microRNAs (miRNAs) or RNA-binding proteins, lncRNAs can impact on mRNA stability, translation, and localization. Acting as miRNA sponges, lncRNAs sequester miRNAs away from their mRNA targets, thereby relieving translational repression and allowing the expression of target genes. Additionally, lncRNAs can guide RNA-binding proteins to specific RNA molecules, thus modulating their processing or stability. Furthermore, lncRNAs play a role in subcellular compartmentalization, shuttling between different cellular compartments and influencing localized processes. This localization-dependent function allows lncRNAs to contribute to spatially restricted activities, such as nuclear organization, RNA trafficking, and protein localization.

Since lncRNAs are so deeply involved in cell physiology, alterations and dysfunctions of their normal activities lead to several types of diseases, including brain disorders in children and adults [1,7,8,9].

### 1.2. Methodological Approaches for the Study of lncRNA Expression and Function

The study of lncRNAs requires a diverse toolkit of techniques and methodologies, ranging from high-throughput sequencing to advanced imaging and computational analyses [10].

Omics techniques, comprising RNA-sequencing and its evolutions, such as global run-on sequencing [11] and native elongating transcript sequencing [12], are crucial for the discovery of new and unannotated lncRNAs. A precise annotation of lncRNA genes is fundamental and not so easy to achieve since they are often monoexonic, non-polyadenylated, or transcribed in antisense direction.

Functional studies of putative lncRNAs involve different loss- and gain-of-function strategies [6]. CRISPR/Cas9-based strategies are a useful tool for the endogenous modulation of lncRNA expression providing information about transcription-dependent mechanisms [13], while knockdown with antisense oligonucleotides (ASOs) or short interfering RNAs (siRNAs) and ectopic overexpression of the mature non-coding transcript allows the determination of whether the lncRNA is a functional RNA molecule. Additional techniques like chromatin RNA immunoprecipitation (ChIRP) [14], RNA antisense purification (RAP) [15], capture hybridization analysis of RNA targets (CHART) [16], RNA immunoprecipitation (RIP) [17] and RNA pulldown [18] help identify interactions between lncRNAs and DNA, RNA, or proteins.

### 1.3. Role of LncRNAS in Neural Development

Neural development is an intricate process by which the nervous system, including the brain and spinal cord, is formed and organized during embryonic and early postnatal stages [19]. During early embryonic development, a flat sheet of cells called the neural plate forms along the dorsal surface of the embryo. This neural plate undergoes a process known as neurulation, in which it folds and fuses to form a neural tube [20]. The neural tube gives rise to the brain and spinal cord, and at the same time, the neural crest cells, located at the edges of the neural plate, migrate to various regions of the body to give rise to a diverse range of cell types, including peripheral neurons, glial cells, and other specialized cell types [21]. Precise spatial and temporal cues guide the differentiation of neural progenitors into distinct types of neurons, establishing the intricate circuitry that underlies sensory processing, motor control, and cognitive functions [22]. Crucial to neural development is the process of synaptogenesis, where neurons form connections with other neurons called synapses, allowing for the transmission of electrical and chemical signals, ultimately shaping the neural circuits responsible for information processing, learning, and memory [23].

**Figure 1:**
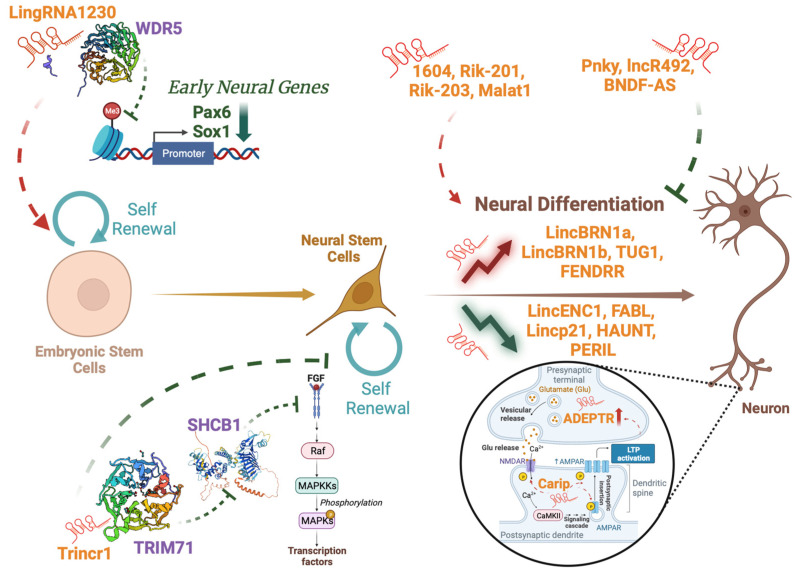
Implications of lncRNAs in neural development. LncRNAs (in orange) can be implicated at multiple steps of neural development, from ESC renewal to the more mature neural phenotype. They can affect target genes (in green) or target proteins (in purple) thus influencing significant processes for neural development.

Throughout neural development, an intricate interplay of genetic and epigenetic factors guides the sequential processes of cell proliferation, differentiation, migration, and circuit formation [24]. Amongst them, lncRNAs were found to play a role in Neural Stem Cell (NSC) proliferation, differentiation, and maturation [25] (Figure 1). Indeed, knockout models of lncRNAs revealed their fundamental implication in brain development [26], and some of these lncRNAs are differentially expressed in both time and space, highlighting a specific function in the brain region where they act [27]. Specifically, the lncRNAs *lincENC1*, *FABL*, *lincp21*, *Haunt* and *Peril* were found to decrease during NSC differentiation, whereas *lincBRN1a*, *lincBRN1b*, *Tug1* and *Fendrr* were found to increase in a contrasting manner [28]. Amongst them, *lincBrn1a* was found to be the most significant, with its impairment resulting in dramatic effects on the developing brain [27].

LncRNAs can regulate the balance between NSC self-renewal and differentiation, influencing the transition of neural progenitor cells from a proliferative state to a differentiated state [25]. They can promote self-renewal acting on both Embryonal Stem Cells (ESCs), as does *LingRNA1230*, which interacts with Wdr5 to inhibit cell transformation into Neural Precursors Cells (NPCs) [29]. The physical interaction between the lncRNA and the protein hinders Wdr5 binding to the promoters of early neural genes such as *Pax6* and *Sox1*, leading to decreased tri-methylation of lysin 4 and 27 of histone 3, thus ultimately resulting in reduced transcription of these genes [29]. The lncRNA *Trincr1* can also impact on self-renewal of both ESCs and NPCs, as it binds to TRIM71 in the cytoplasm, leading to the downregulation of *SHCBP1*, a gene involved in almost all aspects of cancer biology [30], and the subsequent inhibition of the FGF/ERK signaling pathway [31]. Moreover, they can promote neurogenesis (e.g., *RMST*, *Kdm2b*, *Paupar* [32,33,34]), enhance neural differentiation (e.g., *1604*, *Rik-201*, *Rik-203*, *Malat1* [35,36,37]) or, on the contrary, inhibit the process (e.g., *Pnky*, *lncR492*, *BDNF-AS* [38,39,40]). The microenvironment and mechanical stimuli can also impact on gene expression during NSC differentiation, and lncRNAs can also play a role in these mechanisms [41,42,43].

These molecules can also modulate the activity of mature neurons by impacting on neuronal activity and synaptic transmission [8,44,45,46]. They can do this through different mechanisms of action, indicating a complex interplay that can govern neural function. Examples of these mechanisms include the phosphorylation of neural receptors, as does *Carip*, a lncRNA able to affect the phosphorylation of AMPA and NMDA receptors leading to alterations in spatial learning and memory [45]. Recent studies also demonstrated that lncRNAs can be localized at synapses, and that their expression can be modulated in an activity-dependent manner following long-term potentiation, as it happens for the lncRNA *ADEPTR* [46,47]. As lncRNAs can exert such versatile functions in brain development, it is easy to imagine how their disruption could lead to the development of numerous pathological processes.

In this review, we will discuss the most recent findings concerning the pathological role of lncRNAs in pediatric neurological disorders, with the aim of shedding light both on their mechanisms of action and on their potential use in clinic as biomarkers and/or therapeutic targets. Studies cited in this review were selected based on the relevance in the field (using the following key words: brain development, pediatric brain cancer, neurodevelopmental disorders, pediatric neurodegenerative disorders, lncRNAs, lncRNA mechanism, lncRNA biomarker, and lncRNA therapeutic targets), novelty, date of publication (most articles in the range 2018–2023). Only articles from peer-reviewed journals are included.

## 2. LncRNAs in Pediatric Neurological Diseases

**Figure 2:**
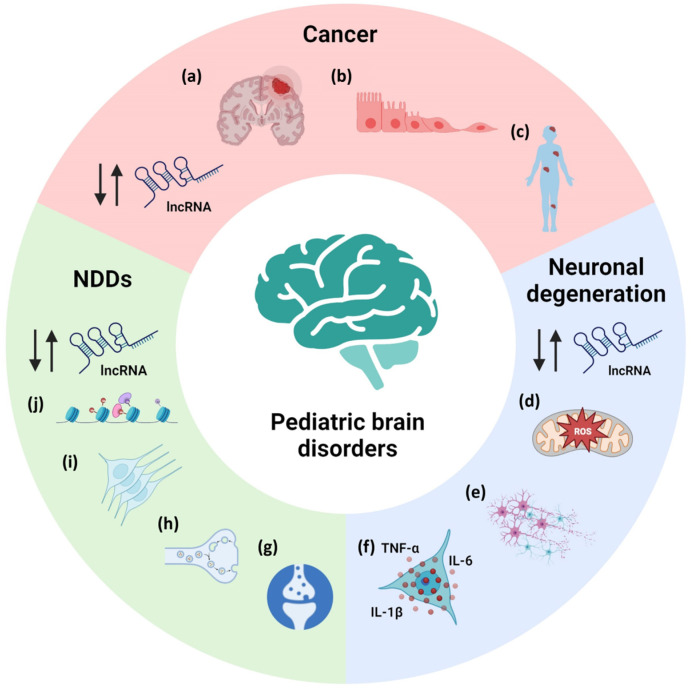
Example of pathological processes controlled by lncRNA dysfunctions in cancer, neurodegenerative disorders and NDDs. (**a**) tumor growth; (**b**) epithelial-to-mesenchymal transition; (**c**) metastasis; (**d**) oxidative stress; (**e**) cell viability; (**f**) inflammation; (**g**) long-term potentiation and long-term depression; (**h**) synaptic vesicle cycle; (**i**) neuronal stem cell proliferation; (**j**) chromatin organization. NDDs: neurodevelopmental disorders. Up and down arrows indicate up- and down-regulation of lncRNA levels.

Pediatric neurological diseases comprise a wide range of pathologies. They can be divided in neurodevelopmental disorders, neurodegenerative disorders and pediatric brain cancers. Despite their intrinsic differences, some pathogenic events are shared, as is the dysregulation of lncRNAs. These lncRNAs were found to act in the most affected pathways in the pathologies, controlling, for example, cell growth and proliferation in cancer, oxidative stress and inflammation in neurodegenerative diseases and stem cell proliferation, and neuronal functions in neurodevelopmental disorders (NDDs) (Figure 2).

### 2.1. LncRNAs in Pediatric Neurodevelopmental Disorders

NDDs refer to a range of disabilities of early onset, which involve a disruption in brain development and ultimately affect cognitive, motor and social-communicative development [48]. These include intellectual disability, communication disorders, Autism Spectrum Disorder (ASD), Attention-Deficit/Hyperactivity Disorder, specific learning disorders and Neurodevelopmental Motor Disorders [49]. Their insurgence can be caused by impairment in multiple pathways, such as cellular metabolism, immune signaling, chromatin remodeling, and synaptic dysfunction [50].

ASD is a complex disease with a yet unknown cause, and in this sense lncRNAs could be new regulators [51]. An interesting study performed in the prefrontal cortex and cerebellum highlighted 200 dysregulated lncRNAs in ASD patients, suggesting potential targets, the function of which is worth being investigated [52]. A characterized lncRNA in the pathology is *SHANK2-AS*, which can form a double-stranded RNA with the mRNA of Shank2, a protein involved in synapse functioning. Indeed, *SHANK2-AS* overexpression was found to impact on neurite complexity and NSC proliferation [53]. Neurological pathways, such as synaptic vesicle cycling, long-term depression and long-term potentiation, were also enriched in a group of 3929 lncRNAs found deregulated in leukocytes of ASD patients [54]. Linking genotype to phenotype could also be important and indeed *MSNP1-AS*, a lncRNA implicated in chromatin organization and immune response, was found dysregulated in individuals carrying the ASD-associated rs4307059 T allele [55,56].

Along with ASD, lncRNAs can be implicated in other NDDs. Fragile X Syndrome is caused by the inactivation or dysfunction of FMR1, required for neuronal connectivity and plasticity [57]. Its gene locus has a bidirectional promoter capable of transcribing the lncRNA *FMR4*, and moreover the lncRNAs *FMR5* and *FMR6* have been recently linked to the disease, all regulators of FMR1 [58,59]. Other examples include *AK081227* and *AK087060*, upregulated in Rett Syndrome brains [60], and *Ube3a-ATS* down-regulated in Angelman Syndrome [61]. All this evidence points to the need for further identification and dissection of lncRNAs in NDDs.

### 2.2. LncRNAs in Pediatric Neurodegenerative Diseases

Pediatric neurodegenerative diseases are rare and devastating conditions affecting children, characterized by progressive nerve cell degeneration [62,63]. Examples of these diseases include Batten disease, metachromatic leukodystrophy, Spinal Muscular Atrophy (SMA) and Niemann-Pick disease (NPD) [64,65,66,67]. Their pathology is varied, but symptoms often include developmental regression, loss of motor skills, muscle weakness, seizures, vision and hearing problems, cognitive decline, and behavioral changes [63]. These disorders are extremely rare and for most of them the implication of lncRNAs is yet to be investigated. Indeed, no evidence is currently available linking lncRNAs with Batten disease and metachromatic leukodystrophy, but indications are emerging connecting these molecules to SMA and NPD [68,69].

SMA is probably one of the most characterized pediatric neurodegenerative diseases, and it is caused by autosomal recessive mutations leading to the deletion of the Survival Motor Neuron 1 (*SMN1*) gene [70]. Recent studies have underscored the pivotal role of lncRNAs in governing SMN protein expression [68]. For instance, *SMN-AS1*, an antisense lncRNA originating from the SMN locus, was found to be a suppressor of *SMN* expression through the recruitment of PRC2 complex [65,71]. Moreover, abnormalities in lncRNA expression were identified at various symptomatic stages of SMA murine models [72]. LncRNAs are also implicated in motor neuron development and muscular atrophy, and this could have strong implications for the pathology of SMA [73,74].

NPD refers to a group of rare and inherited lysosomal storage disorders characterized by the accumulation of lipids, particularly sphingomyelin, within cells [67]. This buildup leads to progressive damage in various organs, including the liver, spleen, and most notably, the nervous system [67]. Symptoms can range from hepatosplenomegaly (enlarged liver and spleen) to neurological manifestations such as developmental regression, loss of motor skills, and intellectual decline [67]. Again, evidence linking lncRNAs to NPD disease is limited, but RNA-sequencing could be helpful towards this goal and one recent study was performed in the cerebellum of a murine model of NPD [69]. This study highlighted 272 dysregulated lncRNAs in NPD, and amongst them, lncRNA *H19* was selected for further characterization [69]. Interestingly, inhibition of this lncRNA was found to reduce ROS levels and improve cell viability, suggesting a functional implication for *H19* in the disease [69].

### 2.3. LncRNAs in Pediatric Brain Tumors

Pediatric brain tumors are the leading cause of cancer-related mortality in children, with a wide spectrum of histological subtypes and clinical behaviors. Despite advances in treatment modalities, the prognosis for many patients remains poor [75]. Hence, there is an urgent need to identify novel therapeutic targets and diagnostic markers to improve patient outcomes. In recent years, there has been a growing interest in the role of lncRNAs in cancer biology, including brain tumors. LncRNAs were shown to regulate gene expression at transcriptional and post-transcriptional levels, influencing various cellular processes such as proliferation, differentiation, and apoptosis [1]. Understanding the functional significance of lncRNAs in pediatric brain tumors may provide valuable insights into their underlying molecular mechanisms and potential as diagnostic or prognostic biomarkers.

Recent investigations have unveiled the dysregulation of numerous lncRNAs in the context of medulloblastomas (MBs). MBs are cerebellar embryonal tumors that comprise 20% of pediatric brain tumors [76]. Enhanced neurosurgery, radiation, and chemotherapy have boosted survival, yet intensive therapies yield long-term neurocognitive, neuroendocrine, and psychosocial issues due to the development of brain vulnerabilities [76]. For instance, overexpression of lncRNA *CRNDE* was found in MB tissues compared to adjacent non-cancerous tissues [77]. Knockdown of *CRNDE* in vitro inhibited colony formation, slowed cell proliferation rates, probably blocking cells in S phase, and promoted apoptosis [77]. Tumor growth was also reduced in vivo upon *CRNDE* silencing and the cancerous tissues from the mouse model showed the decrease in the proliferating cell nuclei antigen (PCNA) and the increase in apoptosis initiator cleaved-caspase-3 [77]. Another study pointed out a possible mechanism of action for *CRNDE* involving the competing endogenous RNA (ceRNA) function; *CRNDE* acts by sponging *miR-29c-3p* [78], which acts as a tumor suppressor gene in different malignancies [79,80,81].

Additionally, lncRNA *linc-NeD125* was notably upregulated in high-grade MBs [82]. In vitro experiments demonstrated its role as a ceRNA, sequestering *miR-19a-3p*, *miR-19b-3p*, and *miR-106a-5p*, thus de-repressing their targets *CDK6*, *MYCN*, *SNCAIP*, and *KDM6A*, known as MB tumor drivers [76]. In accordance, knockdown of *linc-NeD125* in group 4 MB cell model limited cell proliferation, while in group 3 MB cell model its ectopic expression unexpectedly attenuated cell proliferation, migration, and invasion [82]. The authors explained this behavior hypothesizing that the overexpression of *linc-Ned125* increases the G4 driver gene protein products, reducing the aggressiveness of group 3 MBs, known to be highly metastatic tumors. Reduced levels of lncRNA *Nkx2-2as* contribute to tumorigenesis upon constitutive activation of SHH signaling in cerebellar granule cells [83]. Indeed, *Nkx2-2as* functions as a ceRNA, sequestering *miR-103/107* and *miR-548* that target *BTG2/Tis21/PC3* and *LATS1/2* tumor suppressors, consequently its downregulation fosters tumor growth both in vitro and in vivo [83]. Similarly, heightened expression of lncRNAs *UCA1* [84] and *CCAT1* [85] was observed in MB specimens and their silencing significantly impeded MB cell proliferation and migration. Further research by Gao et al. [86] demonstrated that the overexpressed lncRNA *LOXL1-AS1* substantiated its pro-oncogenic role through the PI3K/AKT pathway in MB and that its knockdown attenuates tumor growth, migration and epithelial-to-mesenchymal transition (EMT), promoting apoptosis both in vitro and in vivo.

Another lncRNA, *TP73-AS1*, emerged as clinically relevant in MB, promoting cell survival, migration, proliferation in vitro, and tumorigenicity in vivo [87,88]. Mechanistically, *TP73-AS1* positively regulates *EIF5A2* by sponging *miR-494-3p* [88]. Moreover, suppressing lncRNA *HOTAIR* curbs MB cell proliferation, tumor growth, migration, and invasion while promoting apoptosis via the *miR-1/miR-206-YY1* axis and EMT [89].

Ependymoma (EPN), the third most prevalent pediatric brain tumor, mainly affects children under 5 years of age [90]. This devastating cancer is believed to arise from ependymal cells lining brain ventricles, often in the posterior fossa [90]. Current pediatric EPN treatment entails surgical removal and radiotherapy, yet it hampers patient growth [91]. Genome-wide methylome analysis by Wang et al. [92] revealed lncRNA signatures reflecting tumor histological traits based on lncRNA promoter methylation status. The majority of the identified lncRNAs were hypomethylated and the lncRNA signatures were associated with cancer-related protein-coding genes [92]. Moreover, there are some examples of specific lncRNAs involved in EPN such as *TRERNA*, which, by overseeing the EMT master transcription factor Snail, exhibits marked overexpression in intracranial subgroups versus normal brain [93]. This correlates with elevated proliferation rates and shortened progression-free survival [93]. Additionally, low expression of some of the HOX transcription factor genes and their related lncRNA *HOTAIR* was identified in EPN through transcriptome analysis [94].

Pilocytic astrocytomas (PAs) are prevalent childhood neoplasms within the central nervous system (CNS), constituting around 20% of pediatric brain tumors [95]. These tumors are typically categorized as benign and can manifest across the CNS, often affecting the cerebellum [95]. Particularly noteworthy is the lncRNA *HOTAIR* which demonstrates elevated expression in juvenile PAs compared to other pediatric tumor types [94].

*HOTAIR* and its associated protein-coding gene *HOXC* were also found to be overexpressed in atypical teratoid/rhabdoid tumor (ATRT) tissues [94], a rare neoplasm characterized by heterogeneous cells resembling embryonic and muscular cells [96], although the underlying mechanism needs further investigation.

Diffuse intrinsic pontine glioma (DIPG) is a subtype of advanced grade gliomas that originates in the pons and spreads to other parts of the brainstem [97]. Although a myriad of treatments have been studied in hundreds of clinical trials, no effective cures are available for this type of cancer [98]. Through expression profiling analysis, Liu et al. uncovered new differentially expressed lncRNAs in DIPG, such as *AF086127*, *AF086391*, *AF119852*, *AK021535*, *AK022370*, *AL050068*, *BC012548*, and *BC041658* [99]. These lncRNAs exhibit strong links to DIPG survival, suggesting promising roles as diagnostic or prognostic biomarkers [99].

Finally, glioblastoma (GBM) stands as the prevailing and most severe primary brain tumor in adults, constituting 8–12% of all pediatric CNS tumors. Marked by swift, extensive infiltration and proliferation, GBM is distinguished by substantial cellular diversity, culminating in high resistance to therapeutic interventions. The lncRNA *MALAT1* exhibits elevated expression in GBM, correlating with unfavorable patient outcomes [100,101]. Its action involves ceRNA mechanism regulating miRNAs, *miR-155* [100] and *miR-199a* [101], which significantly influences tumor progression.

Moreover, different studies confirm *HOX* genes and *HOTAIR* dysregulation in pediatric GBMs. Operating as a miRNA sponge, increased levels of *HOTAIR* can, on one hand, favor tumor growth and proliferation [102] and on the other hand, contribute to drug resistance by sponging *miR-125* [103].

There are also examples of lncRNAs that normally act as tumor suppressors, which are downregulated in GBM such as *DGCR5*. Its overexpression causes cell cycle arrest, increased apoptosis and the inhibition of EMT [104].

## 3. Discussion and Conclusions

Mounting evidence strongly supports the involvement of lncRNAs as players in pediatric neurodevelopmental disorders and neuro-oncology. Notably, the early detection of these diseases, especially in very young children, emphasizes the pressing requirement for biomarkers that can offer valuable insights into disease progression and provide non-invasive and sensitive tools for disease detection and monitoring. The unique characteristics of lncRNAs, such as tissue-specific expression patterns and their involvement in disease states, make them attractive candidates as biomarkers for diagnostic and prognostic purposes.

In cancer, the study of lncRNAs could facilitate the identification of molecular markers aiding tumor classification and patient risk assessment, in conjunction with other clinical/biological characteristics. So far, there are only few examples of lncRNAs with potential as biomarkers for brain tumors and none of these studies is specific for pediatric diseases. This is the case of the previously mentioned lncRNA *CRNDE*, the overexpression of which in glioma tissue correlates with a higher grade, tumor volume and recurrence [105]. A signature of multiple lncRNAs has been also proposed as a biomarker for tumor stratification. By comparing the lncRNA profiles in normal, low-grade and high-grade astrocytomas and oligodendrogliomas, Zhang et al. found different sets of lncRNAs specifically associated to malignancy grades [106]. Alongside studies highlighting lncRNAs as potential tissue biomarkers, a report by Tan et al. [107] opened the possibility to use serum-derived *HOTAIR* as a novel prognostic and diagnostic biomarker for GBM; the lncRNA, vehiculated through exosomes, was found overexpressed in serum samples from GBM patients and its sole expression was able to distinguish high-grade vs low-grade brain tumors, as assessed by ROC analysis. Peripheral tissues have been analyzed as a good source of potential biomarkers also in NDDs. As mentioned before, a panel of deregulated lncRNAs was found in leukocytes to discriminate between ASD patients and controls [54].

Besides being possible biomarkers, lncRNAs with key roles in disease could represent novel therapeutic targets. This scenario is supported by several of their characteristics, which at least theoretically match a number of clinical requirements, such as tissue-specificity, fast turn-over and low expression levels. Different approaches are already available for lncRNA targeting, the most advanced of which is represented by ASOs. The first ASOs were single-stranded DNA oligos that bind to their target RNA through Watson–Crick base pairing inducing RNase H-mediated co-transcriptional cleavage at the ASO binding site, ultimately leading to premature transcription termination and reduced lncRNA levels [108,109]. Over the years, chemical modifications of ASOs, such as Locked nucleic acid (LNA)-ASOs, on one hand provided nuclease resistance and improved binding affinity to their target, giving rise to more versatile and less fragile tools, while on the other hand, resulted in different mechanism of action, such as steric block instead of cleavage [110]. ASOs have high transfection efficiency in vitro, but there are some critical points that limit their use in the clinic; firstly the in vivo toxicity, the lack of a proper delivery system, insufficient beneficial effects and off-target side effects. For this reason, studies aimed at improving their pharmacological properties are ongoing, but in the meantime some mRNA-targeting ASOs have already been approved by the Food and Drug Administration and European Medicines Agency, and more advanced clinical trials are underway or are under development [110]. Concerning lncRNA-targeting ASOs, currently there are no ongoing clinical trials (ClinicalTrials.gov). However researchers are exploring this possibility and an interesting example is represented by *BDNF-AS*, antisense to *BDNF* (brain-derived neurotrophic factor), a gene encoding for a protein involved in memory formation [111,112,113]. Interestingly, *BDNF-AS*-targeting antago-Natural Antisense Transcripts (antagoNATs) were successfully delivered across the blood-brain barrier in a murine model, affecting *BDNF* expression in multiple brain regions [111].

Another possible therapeutic strategy is to block lncRNA-protein interaction through small molecules that bind to the target lncRNA or synthetic molecules mimicking the structure and binding properties of lncRNAs, thus working as a decoy. However, this approach is less developed than ASO technology because it requires the identification of relevant RNA motifs with sufficient structural detail [114]. Unfortunately, this level of knowledge is available for a very limited number of lncRNAs. Midway between ASOs and small molecules strategy there is the study by Woo et al. [71], who provided the proof-of-concept of an up-regulation technology based on the use of a chemically modified ASO that acts as a steric blocker. The authors showed that the interaction between *SMN-AS1* and PRC2 could be disrupted in primary neuronal cultures by the modified ASO, thus restoring the expression of *SMN2* gene, usually abrogated in SMA patients [71].

CRISPR-Cas9 system-based methods are emerging as one of the most promising and versatile tools for the precise modulation of specific lncRNAs [13]. The possibility to knockout, knock-down and overexpress a single lncRNA locus is fascinating for therapeutic purposes, however the applications of CRISPR-Cas9 technology in vivo are limited and more challenging as compared to ASOs and small molecules, starting from the proper delivery of all the CRISPR-Cas9 machinery components [115].

Although the translation of lncRNA-based therapeutics to the clinic appears distant, ongoing research holds substantial potential in the realm of pediatric brain disorders. Overall, the number of lncRNAs so far characterized in these diseases is limited, and there is still a need for biomarker and target discovery. For this reason, new studies in affected and peripheral tissues aimed at identifying transcriptional dysregulation in these disorders, namely RNA-sequencing studies, could prove beneficial for the diagnosis and therapy [116]. Oftentimes, these studies are already available, but if lncRNAs are not the primary focus of the work, their investigation might be overlooked. Publicly available datasets could be investigated in this sense, and GEO repositories could be of primary value. Indeed, when searching the GEO database using “Neurodevelopmental Disorders” as term, 580 RNA-sequencing studies were found as deposited online, with 309 being performed in Mus Musculus, 213 in Homo Sapiens, and the remaining in other species such as Rat and Drosophila [106]. From these studies, researchers could filter for the disease of interest and integrate different datasets to obtain solid data on lncRNA expression in NDDs. Interestingly, only 6 datasets have been deposited when searching the term “pediatric neurodegenerative disorders” on GEO database, 3 in humans and 3 in mice [106]. This partially explains the very limited literature concerning lncRNAs in this particular class of diseases and suggests a broad field in which researchers could venture to explain specific pediatric neurodegenerative processes. Lastly, when searching the GEO database with the key word “pediatric brain tumors”, 67 results emerged, with 36 studies performed in Homo Sapiens and 29 in Mus Musculus and the remaining in Danio Rerio and Drosophila [106]. The integration of datasets and the use of already published material would allow for a greater case study and would save time and resources for researchers. Moreover, the development of novel technologies and methodologies, such as high-throughput sequencing and advanced imaging techniques, will undoubtedly enhance our comprehension of lncRNA biology and their potential therapeutic and diagnostic applications.

## Data Availability

Data sharing not applicable.
